# Cardiovascular mortality and exposure to extremely low frequency magnetic fields: a cohort study of Swiss railway workers

**DOI:** 10.1186/1476-069X-7-35

**Published:** 2008-07-01

**Authors:** Martin Röösli, Matthias Egger, Dominik Pfluger, Christoph Minder

**Affiliations:** 1 Institute of Social and Preventive Medicine (ISPM), University of Bern, Finkenhubelweg 11, 3012 Bern, Switzerland; 2 Department of Social Medicine, University of Bristol, Whiteladies Road, Bristol, BS8 2PR, UK; 3 datametrix AG, Zürich, Technoparkstrasse 1, 8005 Zürich, Switzerland; 4 Horten Zentrum, Medical Faculty, University of Zürich, 8091 Zürich, Switzerland

## Abstract

**Background:**

Exposure to intermittent magnetic fields of 16 Hz has been shown to reduce heart rate variability, and decreased heart rate variability predicts cardiovascular mortality. We examined mortality from cardiovascular causes in railway workers exposed to varying degrees to intermittent 16.7 Hz magnetic fields.

**Methods:**

We studied a cohort of 20,141 Swiss railway employees between 1972 and 2002, including highly exposed train drivers (median lifetime exposure 120.5 μT-years), and less or little exposed shunting yard engineers (42.1 μT-years), train attendants (13.3 μT-years) and station masters (5.7 μT-years). During 464,129 person-years of follow up, 5,413 deaths were recorded and 3,594 deaths were attributed to cardio-vascular diseases. We analyzed data using Cox proportional hazards models.

**Results:**

For all cardiovascular mortality the hazard ratio compared to station masters was 0.99 (95%CI: 0.91, 1.08) in train drivers, 1.13 (95%CI: 0.98, 1.30) in shunting yard engineers, and 1.09 (95%CI: 1.00, 1.19) in train attendants. Corresponding hazard ratios for arrhythmia related deaths were 1.04 (95%CI: 0.68, 1.59), 0.58 (95%CI: 0.24, 1.37) and 10 (95%CI: 0.87, 1.93) and for acute myocardial infarction 1.00 (95%CI: 0.73, 1.36), 1.56 (95%CI: 1.04, 2.32), and 1.14 (95%CI: 0.85, 1.53). The hazard ratio for arrhythmia related deaths per 100 μT-years of cumulative exposure was 0.94 (95%CI: 0.71, 1.24) and 0.91 (95%CI: 0.75, 1.11) for acute myocardial infarction.

**Conclusion:**

This study provides evidence against an association between long-term occupational exposure to intermittent 16.7 Hz magnetic fields and cardiovascular mortality.

## Background

Decreased heart rate (beat-to-beat) variability is a well established predictor of cardiovascular morbidity and mortality [1-4]. Based on a laboratory study [5], which showed that heart rate variability is reduced after nocturnal exposure to intermittent 60-Hz magnetic fields Sastre et al hypothesized that long-term exposure to low frequency magnetic fields (ELF-MF) might be associated with acute myocardial infarction and arrhythmia-related deaths [5]. An increased risk of mortality from these causes was subsequently observed in a cohort of electric utility workers [6]. However, since then several cohort studies failed to confirm this finding [7], and a pooled analysis of laboratory studies did not show a consistent effect on heart rate variability [8].

In a more recent sham-controlled double-blind study, Sastre et al [9] observed reduced heart rate variability and decreases in mean heart rate when volunteers were exposed to intermittent magnetic fields of 16 Hz. They speculated that exposure at this frequency may be a more effective stimulus than 60 Hz fields and alter variability via the central nervous system [9]. Indeed, several double-blind laboratory studies demonstrated changes in human brain electrical activity from ELF-MF exposure, in particularly in the alpha frequency band [10].

**Figure 1:**
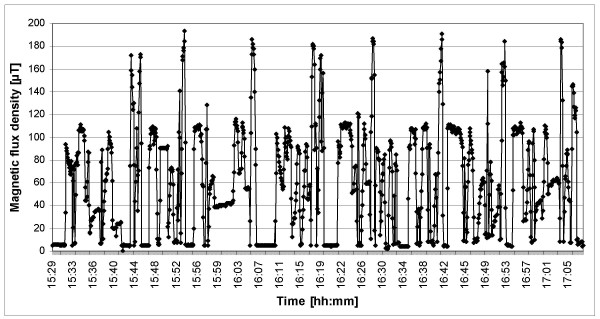
**Magnetic flux density measured on a Swiss express train travelling from Zurich to Bern.** Measurements were taken at the back of the driver's seat in a RE 420 engine.

Railway drivers in some European countries, including Switzerland, Sweden, Norway, Germany and Austria [11], are exposed to intermittent 16.7 Hz magnetic fields. During acceleration and when braking with the engine, magnetic flux density levels can reach several hundred micro-Tesla, whereas ELF-MF are almost negligible during stops in stations (Figure 1). The Swiss Railway Cohort [12-14] therefore offers an unique opportunity to investigate whether long-term exposure to intermittent 16.7 Hz magnetic fields is associated with cardiovascular mortality.

## Methods

### Study population

Detailed descriptions of the cohort and study methods have been published elsewhere [13,14]. In brief, the Swiss Railway Cohort includes all men who were registered as train driver, shunting yard engineer, train attendant or station master in the personnel or pension records of the Swiss Federal Railways between January 1, 1972 and December 31, 2002. Cause of death information was obtained from death certificates. From 1972 to 1994, deaths were coded according to the 8th revision of the International Classification of Diseases (ICD-8), and since 1995, according to the 10th revision (ICD-10). Cause of death information includes initial and consecutive disease as well as a maximum of two concomitant diseases. We determined the cause of deaths by taking into account all available diagnoses. We considered deaths from cardiovascular diseases classified by the following four groups: arrhythmia related diseases, acute myocardial infarction, atherosclerosis related mortality as well as Sub-acute and chronic coronary heart diseases. The corresponding ICD codes are listed in Table 1. We used a probabilistic record linkage method to match employee records with the anonymous death certificates. Records were linked using the software LinkPro 3.0 by an investigator blinded to occupation and exposure status. Linkage variables were date of birth, date of death, place of residence at time of death, occupation, marital status, and duration of marriage, if married. In a first linkage cycle only records with a date of death were considered for the lining. Death certificates were accepted if there was complete agreement on date of birth and death and probabilistic weight calculated from the other linkage variables was positive. In a second cycle records without a date of death were considered and links with death certificates were accepted if the dates of birth matched and the probabilistic weight was at least 40 percent of the maximum weight.

**Table 1:** Cohort characteristics and frequency of deaths among 20,141 Swiss railway workers, by cause and occupational group.

	**ICD-8**	**ICD-10**	**Station** **master**	**Train** **attendant**	**Shunting** **yard** ** engineer**	**Train ** **driver**	**Total**
			N (%)	N (%)	N (%)	N (%)	N (%)
Number of exposed individuals			4,757	6,909	1,378	7,097	20,141
Person years of observation			118,418	160,819	33,752	151,139	464,129
Number of deaths (all causes)			1278 (100%)	2089 (100%)	394 (100%)	1652 (100%)	5413 (100%)
Heart and circulation diseases (all)	390–458	I00-I99	862 (67%)	1368 (65%)	260 (66%)	1104 (67%)	3594 (66%)
Arrhythmia related diseases	427.2, 427.9	I44, I45, I47-49, R00	37 (3%)	76 (4%)	6 (2%)	51 (3%)	170 (3%)
Acute myocardial infarction	410	I21, I22	73 (6%)	125 (6%)	37 (9%)	93 (6%)	328 (6%)
Atherosclerosis related mortality	440	I70	32 (3%)	56 (3%)	9 (2%)	43 (3%)	140 (3%)
Sub-acute and chronic coronary heart disease	411–414	I20, I24 , I25	265 (21%)	444 (21%)	108 (27%)	365 (22%)	1182 (22%)

### Exposure assessment

For each occupational group and year, the average ELF-MF exposure was determined based on measurements and modeling. Cumulative exposure for each individual was obtained by adding up annual workplace specific exposures according to the start and end date of employment obtained from occupational records. An exposure duration of 7.5 hours per day and 240 working days per year was assumed.

A first set of ELF-MF measurements was carried out in 1993–94 using a commercial Gaussmeter (Bramur, Lee, MA) [15]. A second series was done in 2003–04 using an EFA device from Wandel & Goltermann (now Narda Safety Test Solution, Germany) [16]. A 16.7-Hz band pass filter and a sampling interval of 5 seconds (mean RMS values) were used. In train engines, meters were either fixed at the back of the driver's seat or carried as a back-pack. In total, 139 measurement runs were performed in numerous engines under real service conditions, representing 198 operating hours. Past exposure levels of the engines were estimated using the FABEL simulation software [17], which allowed us to take into account factors that have changed for some engines over the study period and that are associated with the magnetic field levels. Such factors included the type of train (freight, normal, or fast train), the driving cycle (e.g. changes in the timetable), and the trainload. For each year, the average exposure at the workplaces of train drivers and shunting yard engineers were separately calculated based on an engine-specific weighted average according to the line and shunting yard engine fleet in service in the respective year. For the period 1919 to 1965, these calculations were done separately for the alpine and the lowland lines because more powerful engines were used in the alpine region, with particularly high field levels at the driver's workplace. Train drivers were classified as alpine or lowland drivers, according to their place of residence.

An additional 41 measurement runs were conducted in passenger coaches and the workplaces of station masters. Average annual exposures for train attendants were derived by calculating weighted means according to the wagon compositions used. Past exposures were corrected for change in the emission from contact wires over time. Based on measurements at several stations, exposure levels of station masters were calculated taking into account their average work time on the platform and in the office.

### Data analysis

We analyzed data using Cox proportional hazards models. The primary time axis was age, period effects were allowed for by splitting the data set into 5-year calendar periods. All models were stratified for the period before and after 1995, thus taking into account the change in coding for cause of death from ICD-8 to ICD-10. Age at entry in the cohort was introduced as a linear, quadratic, and cubic term. We tested all models successfully for the proportionality assumption using Nelson-Aalen survivor functions and statistical tests based on Schoenfeld residuals. Data were analyzed using STATA 10.0 (Stata Corporation, College Station, Texas, USA).

The association between cardiovascular mortality and ELF-MF exposure was modeled using two different exposure metrics. First, the last recorded occupation of each individual served as a proxy for exposure. Cause-specific mortality of highly exposed train drivers as well as medium exposed shunting yard engineers and train attendants was compared with little exposed station masters. Second, individual cumulative exposure, expressed as microtesla-years (μT-years), was entered in the model as a continuous time-varying covariate. We calculated hazard ratios per 100 μT-years increase in cumulative exposure. In addition, we conducted a sensitivity analyses with censored datasets. Among the very old cardiovascular mortality may no longer be affected by occupational exposures and diagnostic accuracy for cardiovascular disease may be reduced. Thus, we censored our observations at the age of 80 years and at the age of 65 years. The latter is the age of retirement for men in Switzerland.

The study was approved by the Federal Commission of Experts for Professional Secrecy in Medical Research.

## Results

### Cohort characteristics

The cohort consists of 20,141 men, with a follow-up of 464,129 person-years (Table 1). Train drivers and train attendants each contributed about one third of the person-time at-risk. In total, 5,413 deaths were observed; 3,594 deaths were attributed to cardio-vascular diseases.

Personnel and pension records contained files for 3406 men with an exact date or year of death. Among these, 76 men (2.2 percent) could not be linked to a death certificate. Most were probably foreigners who returned to their country of origin. These deaths were considered as deaths with unknown cause. A total of 1968 additional deaths were identified among the remaining 16,735 men. None of these matched more than one death certificate. Data from the 39 men found to be older than 100 years at study end (December 31, 2002) were censored at the age of 100 years.

### Exposure assessments

For train drivers, the average exposure at their workplace was approximately 21 μT (Table 2). Shunting yard engineers were exposed to about 6 μT. The exposure of train attendants was below 2 μT up to 1980. Since then, exposure increased to about 4 μT as a result of the introduction of new coaches. The exposure of station masters was around 1.0 μT. The median cumulative lifetime exposure of train drivers was about three times higher than the median exposure of shunting yard engineers, about nine times higher than that of train attendants and 21 times higher than that of station masters (Table 2).

**Table 2:** Average magnetic field exposures among 20,141 Swiss railway workers.

**Exposure measure**	**Station** **master**	**Train** **attendant**	**Shunting ** ** yard** **engineer**	**Train driver**
Average exposure in 1980 [μT]	0.7	1.8	5.5	21.4
Average exposure in 2000 [μT]	1.0	4.2	6.0	21.0
Median cumulative lifetime exposure [μT-years]	5.7	13.3	42.1	120.5

Values are given in μT or μT-years for each occupational group.

### Mortality

All-cause and cardiovascular mortality of train drivers and station masters were similar, but somewhat higher in shunting yard engineers and train attendants (Table 3). For all four diagnostic subgroups similar distributions of the hazard ratios among the four occupations were observed. Hazard ratios for train drivers were close to 1. Significant increased hazard ratios for acute myocardial infarction and for sub-acute and chronic coronary heart disease were observed for shunting yard engineers.

**Table 3:** Hazard ratios (HR) including 95% confidence intervals (95%CI) for various cardiovascular diagnostic groups among 20,141 men Swiss railway workers, by occupational group.

**Outcome**	**Station** ** master ** **(Reference)**	**Train ** **attendant**	**Shunting ** ** yard** **engineer**	**Train driver**
	HR (95%CI)	HR (95%CI)	HR (95%CI)	HR (95%CI)
All-cause mortality	1 (-)	1.13 (1.05, 1.21)	1.12 (1.00, 1.26)	1.02 (0.95, 1.10)
Heart and circulation diseases, all	1 (-)	1.09 (1.00, 1.19)	1.13 (0.98, 1.30)	0.99 (0.91, 1.08)
Arrhythmia related diseases	1 (-)	1.30 (0.87, 1.93)	0.58 (0.24, 1.37)	1.04 (0.68, 1.59)
Acute myocardial infarction	1 (-)	1.14 (0.85, 1.53)	1.56 (1.04, 2.32)	1.00 (0.73, 1.36)
Atherosclerosis related mortality	1 (-)	1.17 (0.76, 1.81)	1.34 (0.63, 2.85)	1.02 (0.65, 1.62)
Sub-acute and chronic coronary heart disease	1 (-)	1.10 (0.94, 1.28)	1.37 (1.09, 1.72)	1.04 (0.89, 1.22)

adjusted for age and 5 year calendar periods as well as stratified for the period before and after 1995, when coding changed from ICD-8 to ICD-10.

There was little evidence for an increase in risk with cumulative exposure. The hazard ratio for arrhythmia related deaths per 100 μT-years cumulative exposure was 0.94 (95%CI: 0.71, 1.24) and 0.91 (95%CI: 0.75, 1.11) for acute myocardial infarction. Censoring our observations at the age of 65 years (Table 4) or 80 years (data not shown) did not produce materially changed hazard ratios.

**Table 4:** Hazard ratios (HR) including 95% confidence intervals (CI) of various cardiovascular diseases among 20,141 men Swiss railway workers with a dataset censored all observations at the age of 65 years.

**Outcome**		**Station** ** master ** **(Reference)**	**Train ** **attendant**	**Shunting ** **yard ** **engineer**	**Train driver**
Heart and circulation diseases, all	No. of deaths	144	217	59	141
	HR (95%CI)	1 (-)	1.19 (0.96, 1.47)	1.25 (0.92, 1.70)	0.95 (0.75, 1.21)
Arrhythmia related diseases	No. of deaths	6	6	2	7
	HR (95%CI)	1 (-)	0.62 (0.20, 1.99)	1.01 (0.20, 5.03)	1.13 (0.37, 3.41)
Acute myocardial infarction	No. of deaths	23	33	14	26
	HR (95%CI)	1 (-)	1.14 (0.67, 1.97)	1.90 (0.97, 3.71)	1.11 (0.63, 1.95)
Atherosclerosis related mortality	No. of deaths	5	1	1	3
	HR (95%CI)	1 (-)	0.16 (0.02, 1.40)	0.72 (0.08, 6.26)	0.63 (0.15, 2.70)
Sub-acute and chronic coronary heart disease	No. of deaths	40	80	20	44
	HR (95%CI)	1 (-)	1.50 (1.02, 2.21)	1.47 (0.86, 2.52)	1.04 (0.67, 1.60)

adjusted for age and 5 year calendar periods as well as stratified for the period before and after 1995, when coding changed from ICD-8 to ICD-10.

## Discussion

### Principal findings

The results from this large cohort study of railway workers in Switzerland do not support the hypothesis that intermittent occupational exposure to 16.7 Hz magnetic fields over many years is associated with mortality from arrhythmia related heart diseases or acute myocardial infarction. This study confirms the largely negative findings from cohort studies of occupations exposed to power frequency magnetic fields (50 or 60 Hz) [6,18-25]. Taken together, the available data from epidemiological studies thus provide little evidence for a link between exposure to magnetic fields and heart disease, over a wide frequency range of 16 to 60 Hz.

### Results in context with other literature

Several double blind studies documented acute effects of ELF-MF on cardiovascular functions under laboratory conditions [5,9,26,27]. These studies provided the biological basis for the hypothesis that long-term exposure to ELF-MF may increase the risk of arrhythmia related events and acute myocardial infarction [5]. However, effects on cardiovascular function were not consistently seen in other studies, including a pooled analysis of seven experiments [8,28]. Also, the heart rate variability of Italian railway drivers during work was not different from that measured in the absence of exposure, 12 hours after the end of the last shift [29]. Data on long-term effects on cardiovascular functions are scarce. Bortkiewicz and colleagues reported decreased heart rate variability in 63 workers of switchyard substations compared to a control group of 42 unexposed radio link workers [30]. Conversely, in a cross-sectional survey of 627 railway high-voltage substation workers no differences in the electrocardiogram were observed between exposed and control groups [31].

Several cohort studies have addressed the question of whether workers exposed to power frequency fields (50 or 60 Hz) are at increased risk of dying from cardiovascular diseases. In one of the first epidemiological studies of power frequency magnetic fields, Savitz et al observed an increase in arrhythmia related deaths [6]. Most [18,20-24], but not all [19,25,32], of the later studies did not confirm these findings. Kheifets and colleagues [7] recently reviewed the literature, observing that in the study by Savitz et al [6], the excess in acute cardiovascular deaths was accompanied by a deficit of deaths from chronic cardiovascular conditions. Also, they argued that the inability to control for potentially important lifestyle-related factors, such as smoking or physical activity could explain the associations found in some studies [7]. Kheifets et al concluded that taken together the evidence speaks against an etiologic relation between exposure to electric and magnetic fields and cardiovascular disease [7].

### Strengths and limitations

The assessment of long-term exposure to ELF-MF is challenging, and an important weakness of many epidemiological studies [7]. In this context our study population is particularly appealing. Swiss railway employees are generally employed long-term, with limited job changes [13,14]. Exposure levels vary greatly across different occupations, with train drivers being exposed to very high ELF-MF levels whereas exposure of station masters is comparable to the general population. In our study, the exposure contrasts between highly and lowly exposed individuals was considerably larger than in previously published occupational cohorts. The exposure circumstances of railway workers are well defined, which allowed the effective magnetic flux density to be measured during work condition as well as modeling of past exposures. Detailed company registers reduced the potential for selection bias and allowed assessments of ELF-MF exposure that are based on individual job histories. Exposures to chemicals or electric shocks, which are frequent in other occupational settings, for example in electric utility workers or welders, are rare in railway workers. Finally, the large size of the cohort and the long follow-up period of 31 years are other important strengths.

The use of outcome data derived from death certificates is a limitation our cohort shares with other studies of cardiovascular mortality [7]. Diagnoses on death certificates have been shown to be inaccurate to some extent [33]. Inaccurate coding of cause of deaths is expected to be unrelated to exposure and would therefore create non-differential outcome misclassification resulting in an underestimation of any outcome-exposure association, if such an association is present. Some non-differential misclassification might also have been introduced by the probabilistic record linkage, which was used to obtain cause-of-death information. However, all hazard ratios for train drivers were close to 1 and an important risk increase could only have been missed if major outcome misclassification had occurred, which is unlikely.

We controlled for potential confounding from age and time trends but we had no data about individual cardiovascular risk factors which may have acted as confounders in these analyses, for example smoking and levels of physical activity. A survey of 378 railway employees carried out in 1994 found that station masters and train drivers were less likely to smoke (8% and 12%, respectively) than shunting yard engineers and train attendants (38% and 29%, respectively) [12]. Socio-economic status showed a corresponding pattern: the salary is similar for train drivers and station masters but somewhat lower for shunting yard engineers and train attendants. In our view, lifestyle factors are probably responsible for the generally increased cardiovascular mortality of shunting yard engineers and train attendants compared to station masters and train drivers. However, the similar socio-economic status of train drivers and station masters and the mortality patterns observed make it unlikely that confounding factors have masked an association between cardiovascular diseases and ELF-MF.

## Conclusion

Our data provide evidence against an association between long-term exposure to 16.7 Hz magnetic fields and cardiovascular mortality, including arrhythmia related deaths or deaths from acute myocardial infarction. These results are in line with most epidemiological studies on exposure to power frequency magnetic fields although we investigated another frequency of the magnetic field spectrum and dealt with higher exposure levels than previous studies. The results from our study suggest that previous negative findings can be generalized to higher exposure levels and to a wider frequency range of the extremely low frequency spectrum including 16.7 Hz.

## Competing interests

The authors declare that they have no competing interests.

## Authors' contributions

CM initiated the first Swiss railway cohort study. MR conceived the idea of the follow-up study and designed the study. ME, DP and CM contributed to the design and coordination of the follow-up. DP performed ELF-MF measurements and prepared the cohort data. MR performed the statistical analysis. MR drafted the manuscript with contributions from all other authors. All authors read and approved the final manuscript.
